# Frozen algorithms: how the brain's wiring facilitates learning

**DOI:** 10.1016/j.conb.2020.12.017

**Published:** 2021-04

**Authors:** Dhruva V Raman, Timothy O’Leary

**Affiliations:** Department of Engineering, University of Cambridge, United Kingdom

## Abstract

Synapses and neural connectivity are plastic and shaped by experience. But to what extent does connectivity itself influence the ability of a neural circuit to learn? Insights from optimization theory and AI shed light on how learning can be implemented in neural circuits. Though abstract in their nature, learning algorithms provide a principled set of hypotheses on the necessary ingredients for learning in neural circuits. These include the kinds of signals and circuit motifs that enable learning from experience, as well as an appreciation of the constraints that make learning challenging in a biological setting. Remarkably, some simple connectivity patterns can boost the efficiency of relatively crude learning rules, showing how the brain can use anatomy to compensate for the biological constraints of known synaptic plasticity mechanisms. Modern connectomics provides rich data for exploring this principle, and may reveal how brain connectivity is constrained by the requirement to learn efficiently.

## Highlights

• Theory tells us why learning is hard to implement in the brain using local plasticity mechanisms.
• These difficulties can be overcome by feedback pathways and redundant connectivity.
• Connectomics reveals such connectivity motifs in learning centers across species.

**Current Opinion in Neurobiology** 2021, **67**:207–214

This review comes from a themed issue on **Neurobiology of learning and plasticity**

Edited by **Tara Keck** and **Sheena A Josselyn**

For a complete overview see the Issue and the Editorial

Available online 25th December 2020

**https://doi.org/10.1016/j.conb.2020.12.017**

0959-4388/© 2021 The Author(s). Published by Elsevier Ltd. This is an open access article under the CC BY license (http://creativecommons.org/licenses/by/4.0/).

## Introduction

The brain learns by modifying itself in response to information from the external world. Since the earliest experimental demonstration of how learning can be implemented by synaptic modification [1,2], experimental attention has focused on mechanisms that link experience-dependent signals to modifications in synaptic strength. Over many decades of research we have witnessed an explosion of different synaptic learning rules, from timing-dependent mechanisms, to neuromodulator induced plasticity as well as combinations of both [3,4].

Paradoxically we are still unable to connect these many kinds of synaptic modification mechanisms to high-level biological learning at the circuit or behavioral level. Basic theory from optimization sheds light on the problem of connecting biological learning to low-level plasticity rules [5^••^,^6^,^7^,8^••^,9^•^,^10^,11^•^]. A key insight is the so-called *credit assignment problem*. In a large network with many interconnected cells there is a complex relationship between a change in any given connection and a change in overall behavior. This difficulty means that each synapse may require detailed information in order to modify in the correct way during learning [12^••^].

Recent progress in AI shows that conceptually simple optimization processes can allow artificial neural networks to achieve superhuman performance on cognitive tasks. The high-level design of such AI systems has informed systems-level hypotheses on biological learning (e.g. [13]). However, the training step requires exchange across the network of vast amounts of information, depending upon the state of each synapse.

To a greater extent than artificial neural networks, biological neural circuits face limiting constraints on the speed, accuracy, and quality of information that can be exchanged between synapses and from the external world. This limits the set of plausible learning mechanisms, and, we argue here, imposes heavy constraints on circuit architecture that are beginning to be revealed in recent connectomics work [14^••^,15^••^, 16].

We will outline key theoretical aspects of learning from an optimization perspective. This will give a concrete framework for understanding why learning is hard computationally, and how this problem can be partially solved with specific circuit connectivity. This topic does not address all aspects of learning, nor does it address all ways in which circuit architecture aids or hinders learning. We will cite recent experimental connectomics work that provides striking examples of circuit motifs that make sense in light of concrete theory. We will also review recent theoretical work that shows how circuit architecture can aid learning, providing hypotheses for future experimental studies.

## Theoretical background: why is learning hard in large neural circuits?

Consider a neural circuit with N synapses. Abstractly, learning minimises some measure of behavioural error (a ‘loss function’) that depends on all N synapses. Any change in the synapses can be represented as an N-dimensional vector, endowing collective synaptic changes with a direction and a magnitude. The credit assignment problem boils down to three general factors that determine the difficulty of learning:

Factor 1 The sensitivity of behavioural performances to ‘good’ synaptic changes.
Factor 2 The robustness of behavioural performance to ‘bad’ synaptic changes.
Factor 3 The relative proportions of ‘good’ and ‘bad’ changes induced by a synaptic learning rule.

What do we mean by ‘good’ and ‘bad’ changes? At any point in time during learning, there is a direction of maximal sensitivity of the loss function to changes in the synapses. This corresponds to the gradient of the loss function, which defines the ‘good’ direction of change.

Artificial learning algorithms compute gradients of loss functions directly to solve the credit assignment problem. In optimization theory, this is known as a ‘first-order’ method [17], which explicitly computes the sensitivity of a circuit-level property to a change in synaptic weights, before adjusting all weights collectively to steer the network to a desired state. However, computing the full gradient generally requires knowledge of all synaptic weight values. While possible on a computer, there is no feasible means of exchanging this amount of weight information explicitly in the brain [12^••^].

Biologically, this means that synaptic changes may only correlate with the gradient, with some proportion of synaptic changes that are not related to the gradient. These correspond to ‘bad’ changes.

An example of a class of biologically plausible learning rules that suffer from a problematic proportion of bad changes is as follows. Apply a random perturbation to all N synapses, then retain the perturbation or reverse it depending on the observed effect on behavioural performance. It is easy to see how a biological synapse might implement such a rule: all it requires is access to the recent error history, for example by integrating a global neuromodulatory signal over time. Learning rules of this form are known as ‘zero-order’, as they do not directly approximate the gradient. Examples include the MIT rule [18], the REINFORCE algorithm [19], and node perturbation [19,20]. Biologically plausible implementations have been proposed as the underlying mechanisms for birdsong learning [21] and cerebellar learning [22^••^].

Unfortunately, although plausible biologically, such learning rules do not work well if they operate on large populations of synapses. A calculation outlined in Box 1 shows that the correlation of such learning rules with the gradient drops in proportion to $1/\sqrt{N}$ where N is the number of synapses. Put another way, biologically plausible zero-order rules only provide feedback on a single direction in weight space, while the full gradient provides information on all directions. Intuitively, we can see that as N increases, the chance that a random direction in weight space aligns with the gradient drops. For zero-order rules this means that the proportion of bad synaptic changes is likely to grow with network size. There are two ways to mitigate this problem:

Box 1 **Mathematical analysis of learning**

Denote behavioural performance in a task by a loss function L[w], where w is the N-dimensional vector of synaptic weights in a network. Learning involves descending this loss function. ‘Gradient-based’ learning rules explicitly form an approximation of the negative gradient −∇L[w(t)] and adjust synaptic weights in this direction during learning. However, *any learning rule*, even ‘gradient-free’ learning rules, must adjust synapses in direction that anticorrelates with ∇L[w] on average. Consider a small time interval δt, in which the weights change by an amount δw. We can write the change in behavioural error over time t+δt as

(1) $$L[\text{w}+\delta \text{w}]-L[\text{w}]\approx \nabla L{\left[\text{w}\right]}^{T}\delta \text{w}+\frac{1}{2}\delta {\text{w}}^{T}\nabla^{2}L[\text{w}]\delta \text{w},$$

where ∇L[w] is the gradient of the loss function, and $\nabla^{2}L[\text{w}]$ is the Hessian (second derivative). If learning occurs in δt, the change in error in 1 will be negative. First, consider the average effect of a completely random weight change, δw, on loss. In this case,

$$\text{E}[L[\text{w}+\delta \text{w}]-L[\text{w}]]=0+\frac{1}{2}∥\delta \text{w}{∥}_{2}^{2}\frac{\mathrm{Tr}(\nabla^{2}L[\text{w}])}{N},$$

where Tr(•) is the trace of a matrix (sum of eigenvalues). We can see that

• A random perturbation is uncorrelated, in expectation, with the gradient. So the first term on the RHS is zero.
• Therefore $\frac{\mathrm{Tr}(\nabla^{2}L[\text{w}])}{N}$ quantifies the robustness of the learning system to random perturbations (Factor 2 in the main text).
• In a partially trained network, random perturbations are expected to negatively affect behavioural performance, that is, $\mathrm{Tr}(\nabla^{2}L[\text{w}])>0$.

Now consider a perturbation-based (0 order) learning rule. Let δw be a random Gaussian perturbation that is kept or reversed so that it anticorrelates with the gradient. In this case, $\delta {\text{w}}^{T}\nabla L[\text{w}]$ has a half-normal distribution. In expectation, the first term of the RHS of Equation 1 reduces to

(2a) $$\text{E}[\delta {\text{w}}^{T}\nabla L[\text{w}]]=\sqrt{\frac{2}{N\pi }}∥\delta \text{w}{∥}_{2}∥\nabla L[\text{w}]{∥}_{2},$$

Thus the correlation with the direction of steepest descent decreases at a rate proportional to $\frac{1}{\sqrt{N}}$. At the other extreme, exact (batch) backpropagation uses the update

δw=−c∇L[w],

for some constant of proportionality c. In this case, the correlation with the gradient is

(2b) $$\text{E}[\delta {\text{w}}^{T}\nabla L[\text{w}]]=-c∥\nabla L[\text{w}]{∥}_{2}^{2}.$$

In either case, $∥\nabla L[\text{w}]{∥}_{2}$ defines the sensitivity of behavioural performance to plasticity in ‘good’ directions, and is a mathematical analogue to Factor 1 in the main text.

Intermediately to Equations 2a and 2b, a noisy first-order rule might align only a small amount with the gradient, like 2a, but have a constant degree of alignment that is independent of N, like 2b. For these learning rules (or for anywhere $\text{E}[\delta {\text{w}}^{T}\nabla L[\text{w}]]$ degrades at a slower rate than $\frac{1}{\sqrt{N}}$) adding apparently redundant neurons can increase the speed and eventual degree of learning [5^••^].

Alt-text: Box 1

(1) Increase the dimensionality of feedback signals. A better correlation between weight changes and the full gradient can be achieved if the feedback signal itself has multiple components. This can be achieved in the brain via multiple, independent feedback pathways that carry vectorised error information to neural circuits. Biologically, error signals can be encoded in neural activity and, in particular, in neuromodulatory signals that convey behavioural valence directly to synapses where learning occurs. We will review recent connectomic studies, particularly in invertebrates, that have uncovered the existence of such precise modulatory feedback in learning centers in the brain.

(2) Use circuit architectures that are robust to ‘bad’ perturbations (i.e. improve Factor 2), while maintaining sensitivity to the ‘good’ component (i.e. maintain Factor 1). It turns out, perhaps surprisingly, that redundant connectivity in a neural circuit can reduce behavioural sensitivity to bad synaptic changes while maintaining sensitivity to good changes. This means that even crude but biologically plausible synaptic learning rules may achieve efficient learning with the appropriate circuit architecture.

In the following sections we review recent work that suggests these principles are at work in the brain.

## Connectomic evidence for vectorised learning signals in the brain

Imagine repetitively practicing and attempting to improve a tennis serve. A coach evaluates each serve with a mark out of 10. This is an example of *scalar* feedback, since the entire behaviour is reduced to a single number. With scalar feedback and multiple behavioural parameters, it is hard to determine which behavioural aspects should be altered, and how.

Now suppose you can correlate aspects of the behaviour (the serve) to the coach's mark, and gradually build up a picture of which behavioural aspects contribute to a good mark (hit the ball harder, keep head raised higher, etc.). Such multifaceted error information is an example of *vector feedback*.

**Figure 1:**
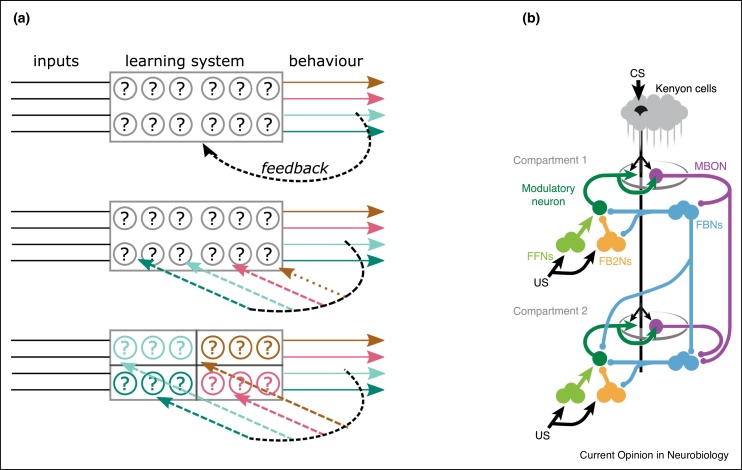
**(a)** Schematic of a learning circuit with four behavioural outputs (denoted by colour) and feedback signals targeting connections that adapt during learning. Each row depicts a feedback signal with different degrees of coarseness. **Top:** A scalar (coarse) feedback signal provides information on overall behavioural performance to all connections. In this scenario the credit assignment problem is most onerous because the individual contributions of circuit connections to behavioral performance are hard to disentangle. **Middle**: A more detailed vector feedback signal specifies how changes in each of the behavioural outputs contributes to overall performance. **Bottom:** Separate subsets of the learning system inform separate behavioural outputs. Now vector feedback helps even a perturbation-based learning rule, as the number of synapses per behavioural output decreases by a factor of four. **(b)** Schematic wiring diagram of the extended MB circuit taken from [13^••^]. Separate compartments are innervated by separate neuromodulators encoding distinct forms of feedback on behavioural performance.

Figure 1a depicts the architecture of feedback signals in a generic neural circuit, from a coarse (scalar) feedback error signal to a fine, vector signal that decomposes error across different behavioural readouts and/or subsets of circuit connections. Higher dimensional feedback eases the difficulty of learning through factor 3. This is because the more connections that are affected by a given component of an error signal, the worse the ratio of ‘good’ changes to ‘bad’ changes becomes (see Equation 2.

Regardless of the specific learning mechanism, more targeted information on behavioural contributions to a task cannot be a bad thing. This benefit can be enhanced further by the partitioning of subcircuits according to the behaviour they influence. For example, suppose separate neural subcircuits control head direction and racket force during the tennis serve in the example above. If these behavioural variables are uncorrelated and targeted feedback on how to change each variable reaches separate neural circuits, then the credit assignment problem need only be solved in each separate subcircuit. On the other hand if head direction and racket force influence each other than this benefit disappears, as both subcircuits collectively influence both behavioural variables.

There is a large body of literature detailing how abstract properties of behaviour, such as reward prediction error, are represented in the brain and inform learning [3,5,^23^,^24^]. Minimally, such error information can correspond to a scalar error signal from bulk release of neuromodulators such as dopamine.

However, more recently, detailed connectomics in *Drosophila* suggests that evolution has exploited the advantages of vector neuromodulatory feedback for learning. Feedback pathways necessary for learning target discrete neural subcircuits, each responsible for different aspects of behaviour. Moreover, these separate pathways seem to provide weakly correlated signals.

The *Drosophila* mushroom body (MB) mediates associative learning across sensory modalities, especially olfaction. This circuit has an architecture that enables weighted combinations of stimuli, such as a combination of odour signals, to become associated with behavioural actions in an experience-dependent manner [25,26]. Distinct modulatory subpopulations represent different error signals. For example, the optogenetic activation of specific modulatory subpopulations, in concert with an odour stimulus, is sufficient to form an aversive or appetitive association [27].

Even for a particular valence (e.g. appetitive), distinct modulatory subpopulations encode a more finely tuned, multidimensional reward signal. As an example, [28^•^] showed that different subsets of modulatory neurons were required for the associative learning of an odour with two different kinds of reward (fructose versus aspartic acid), even while both triggered the same behaviour of learned odour attraction.

**Figure 2:**
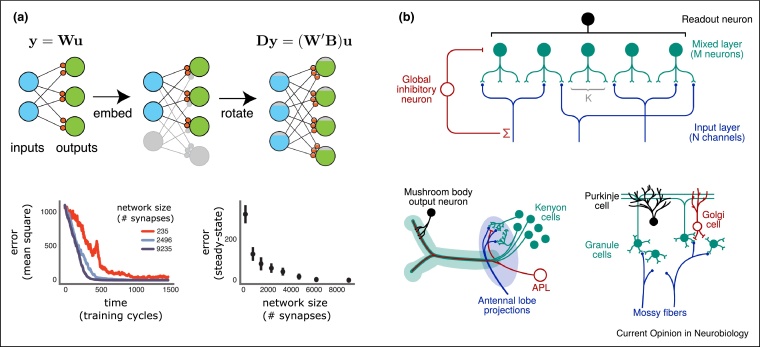
**(a)** Embedding an input-output mapping into a higher dimensional space reduces the difficulty of feedback based learning. Noisy gradient descent, with a fixed signal:noise ratio, will learn faster and to higher steady state performance in a higher dimensional network. The effect is not limited to the particular linear network architecture shown, but also holds for multilayer, nonlinear networks. Figure reproduced from [5^••^]. **(b)** A redundancy enhancing neural circuit motif studied in [8^••^], and its correspondence with biological circuits in the *Drosophila* mushroom body and mammalian cerebellar cortex, respectively. Inhibitory neurons and sparse input connectivity both maximise the representational dimension of the network, allowing for more efficient pattern separation. Figure reproduced from [8^••^].

Is the functional specificity of these modulatory neurons encoded anatomically? Evidence points to different modulatory neurons receiving different sets of sensory afferents, and therefore potentially computing somewhat independent error signals. The mushroom body has a segregated architecture consisting of largely independent neural subpopulations learning associations from different neuromodulatory feedback signals. In [29], the MB was conceptually divided into 15 compartments, defined by the presence of specific dopaminergic (modulatory) and output neuron cell types. Tracing studies including [14^••^,15^••^] found that each modulatory neuron received a unique combination of sensory afferents, with same-type modulatory neurons receiving more similar sensory input. Moreover, many modulatory neurons receive over half of their dendritic input from recurrent connections [14^••^]. This recurrence links different MB compartments, such as those responsible for aversive and appetitive associations (see Figure 2b), thus allowing for reward signals incorporating both sensory input and integrated past experienced from multiple associative systems. The recurrent input is highly diverse [15^••^] across different modulatory neuron types, emanating from different regions including gustatory interneurons and the lateral horn.

Together, this work supports a hypothesis that different neuromodulatory pathways are affected by different aspects of past experience, as well as unique combinations of sensory stimuli. This may provide non-redundant, vector feedback signals to independent neural subpopulations in the MB. This circuit architecture is expensive in terms of developmental complexity and wiring cost, yet the theory discussed in the previous section tells us that this price may be necessary for efficient learning. It would be interesting, on the other end, to see whether different neuromodulatory pathways are associated with the formation of distinct behavioural responses.

We have reviewed the anatomical features in the *Drosophila* mushroom body that could ease the burden of the credit assignment problem. This was made possible by the detailed connectomic information available on the *Drosophila*. The existence of zero-order learning mechanisms has been proposed in higher organisms as well, notably the song-learning system in Zebra finches [16,21,30]. The songbird provides an excellent model system for a difficult learning problem because learning to mimic and recognise birdsong corresponds to learning complex temporal sequences of muscle movements and auditory stimuli: a rich feedback signal. A proposed implementation of local credit assignment, using the node-perturbation algorithm [21] lead to specific anatomical predictions subsequently verified by connectomics studies [16].

## Redundant connectivity can boost learning efficiency of noisy, local synaptic plasticity mechanisms

Clearly, the difficulty of a specific learning problem is influenced by the architecture of the neural circuit used to solve it. We now review some recent theoretical results that provide mechanistic reasons as to why particular, observed architectures are favourable for the learning problems they solve.

One generic architecture that recurs across organisms is the ‘input expansion’. Here a large, horizontal ‘mixed-layer’ of cells receives low numbers of inputs, and projects to low numbers of outputs. For example, this architecture occurs in Kenyon cells in the insect mushroom body, or cerebellar granule cells. One advantage of such an architecture is that it can ‘separate out’ correlated inputs by embedding them in a higher dimensional space, as proposed decades ago in [31]. Recent modelling work has justified more detailed aspects of these architectures [6,7,8^••^]. In [8^••^], a simplified circuit model of the input expansion motif was constructed. The sparsity (i.e. number of inputs projecting onto each mixed-layer neuron), and degree of feedforward inhibition onto the mixed layer were set as tunable parameters, as was the number of neurons in the two layers. They investigated how these tunable parameters related to dimensionality of the representation. This latter quantity is important as the higher it is, the greater the efficacy of associative learning using a Hebbian readout neuron. Optimal sparsity of the input connections to the mixed layer was calculated, and found to correspond exactly to the degree of anatomically observed sparsity in the mossy fibre inputs to cerebellar granule cells to the human brain, as well as antennal lobe inputs to kenyon cells in *Drosophila*.

We have discussed how a particular anatomical circuit motif supports pattern separation, an unsupervised learning problem. The specific anatomical predictions of [8^••^] did not apply in the case where synapses were subject to supervised plasticity, although it was noted that dense connectivity seemed more supportive of fast learning in this case. More recently, [5^••^] showed quantitatively how and why adding neurons in densely connected circuits could speed learning, even when smaller circuits could theoretically solve the learning problem to perfect accuracy. Thus, adding ‘redundant’ neurons can compensate for an inaccurate learning rule that cannot induce plasticity well-aligned with the gradient. The key insight was that adding redundant neurons could increase sensitivity of behavioural performance to ‘good’ directions of plasticity, without decreasing the robustness of behavioural performance to ‘bad’ directions. Mathematically, the sensitivity of behavioural performance corresponded to the magnitude of the gradient, and the robustness depended upon the eigenvalues of the Hessian (see Box 1 for insight and some details). Note that this benefit of redundancy is counteracted, for zero-order learning rules, by the increased burden of the credit assignment problem in large networks (i.e. inability to accurately find the gradient).

The result of [5^••^] suggests that learning rules could be quantified not only by how well they perform in *in silico circuits*, but on how their performance scales as network size increases to biologically realistic levels. A given learning rule may provide a highly inaccurate approximation of the gradient. Nevertheless, if the quality of this approximation does not decrease with increasing network size (or decreases more slowly than zero-order, perturbation-based learning rules), then the described benefits of redundantly large circuits could compensate to nevertheless enable fast, accurate learning. However, these benefits might only emerge at extremely large network sizes.

The previous point is relevant given the plethora of proposed, biologically plausible approximations to backpropagation that have emerged in the literature [12^••^]. Backpropagation itself requires individual synapses to know the states of their downstream counterparts. This is known as the ‘weight transport’ problem. Various biologically plausible surrogates to weight transport partially circumvent this. One solution is to demand parallel feedback connections that instead provide synapses with information on downstream neural activities, which serves as a rough proxy [32, 33, 34, 35, 36, 37, 38, 39, 40, 41]. It is worth noting that these feedback connections effectively provide an error signal with vectorised information on the errors of different output neurons. Another solution is to let individual neurons take the role of an abstract ‘network’, with internal ‘layers’ [9^•^]. This makes weight transport an intracellular problem, and allows for the separation of feedback pathways respectively providing reward and weight transport signals [10,11^•^,^42^].

All of these proposed algorithms explicitly, albeit inaccurately, compute the sensitivities of behavioural performance to individual synapses. This inaccuracy has led to mixed success when benchmarking their performance against artificial learning rules not subject to biological plausibility constraints [43^••^]. However, the results of [5^••^] suggest that learning performance could improve as network size increases to biological levels.

## Conclusion

It is difficult, if not impossible, to understand how synaptic-level learning rules enable behavioural level learning without studying the structure of neural circuits and the abstract mathematical nature of learning as an optimisation process. Even with intricate synaptic plasticity mechanisms, there is a fundamental algorithmic difficulty in implementing learning at the whole circuit or behavioural level due to the credit assignment problem. We have reviewed two very plausible classes of solutions to this problem: vector feedback and redundant connectivity.

Recent advances in quantitative neuroanatomy [25,44, 45, 46, 47, 48] have made it possible to measure and quantify circuit architecture, revealing striking evidence that these solutions are used biologically. We need principles to make sense of the mass of complex data that connectomics reveals. The question of how learning might work algorithmically was successfully addressed long before the era of modern connectomics in Marr and Albus's groundbreaking analysis of cerebellar architecture [31,49]. New theoretical insights and insights from artificial learning, coupled with extraordinarily detailed and comprehensive connectomics data, places us in an exciting era that will leverage experiment and theory to understand biological learning.

## Conflict of interest statement

Nothing declared.

## References and recommended reading

Papers of particular interest, published within the period of review, have been highlighted as:

• of special interest
•• of outstanding interest

## References

[1] Pinsker H., Kupfermann I., Castellucci V., Kandel E.R. (1970). Habituation and dishabituation of the GM-Withdrawal reflex in Aplysia. Science.

[2] Castellucci V., Kandel E.R. (1976). Presynaptic facilitation as a mechanism for behavioral sensitization in Aplysia. Science.

[3] He K., Huertas M., Hong S.Z., Tie X.X., Hell J.W., Shouval Ha., Kirkwood A. (2015). Distinct eligibility traces for LTP and LTD in cortical synapses. Neuron.

[4] Brzosko Z., Zannone S., Schultz W., Clopath C., Paulsen O. (2017). Sequential neuromodulation of Hebbian plasticity offers mechanism for effective reward-based navigation. Learning.

[5••] Raman D.V., Rotondo A.P., O’Leary T.S. (2019). Fundamental bounds on learning performance in neural circuits. Proc Natl Acad Sci U S A.

[6] Babadi B., Sompolinsky H. (2014). Sparseness and expansion in sensory representations. Neuron.

[7] Cayco-Gajic A.N., Clopath C., Silver A.R. (2017). Sparse synaptic connectivity is required for decorrelation and pattern separation in feedforward networks. Nat Commun.

[8••] Kumar A.L., Harris K.D., Axel R., Sompolinsky H., Abbott L.F. (2017). Optimal degrees of synaptic connectivity. Neuron.

[9•] Richards B.A., Lillicrap T.P. (2019). Dendritic solutions to the credit assignment problem. Curr Opin Neurobiol.

[10] Guerguiev J., Lillicrap T.P., Richards B.A. (2017). Towards deep learning with segregated dendrites. eLife.

[11•] Sacramento J., Costa R.P., Bengio Y., Senn W. (2018). Dendritic cortical microcircuits approximate the backpropagation algorithm. Advances in Neural Information Processing Systems 31.

[12••] Lillicrap T.P., Santoro A., Marris L., Akerman C.J., Hinton G.E. (2020). Backpropagation and the brain. Nat Rev Neurosci.

[13] Wang J.X., Kurth-Nelson Z., Kumaran D., Tirumala D., Soyer H., Leibo J.Z., Hassabis D., Botvinick M. (2018). Prefrontal cortex as a meta-reinforcement learning system. Nat Neurosci.

[14••] Eschbach C., Fushiki A., Winding M., Schneider-Mizell C.M., Shao M., Arruda R., Eichler K., Valdes-Aleman J., Ohyama T. (2020). Recurrent architecture for adaptive regulation of learning in the insect brain. Nat Neurosci.

[15••] Otto N., Pleijzier M.W., Morgan I.C., Edmondson-Stait A.J., Heinz K.J., Stark I., Dempsey G., Ito M., Kapoor I., Hsu J., Schlegel P.M., Bates A.S., Feng L., Costa M., Ito K., Bock D.D., Rubin G.M., Jefferis G.S.X.E., Waddell S. (2020). Input connectivity reveals additional heterogeneity of dopaminergic reinforcement in Drosophila. Curr Biol.

[16] Kornfeld J., Januszewski M., Schubert P., Jain V., Denk W., Fee M.S. (2020). An anatomical substrate of credit assignment in reinforcement learning. bioRxiv.

[17] Polyak B.T. (1987). Introduction to Optimization.

[18] Whitaker H.P. (1959). An Adaptive System for Control of the Dynamics Performances of Aircraft and Spacecraft.

[19] Williams R.J. (1992). Simple statistical gradient-following algorithms for connectionist reinforcement learning. Mach Learn.

[20] Flower B., Jabri M. (1993). Summed weight neuron perturbation: an O(n) improvement over weight perturbation. Neural Information Processing Systems.

[21] Fiete I.R., Fee M.S., Seung H.S. (2007). Model of birdsong learning based on gradient estimation by dynamic perturbation of neural conductances. J Neurophysiol.

[22••] Bouvier G., Aljadeff J., Clopath C., Bimbard C., Ranft J., Blot A., Nadal J.-P., Brunel N., Hakim V., Barbour B. (2018). Cerebellar learning using perturbations. eLife.

[23] Schultz W. (2015). Neuronal reward and decision signals: from theories to data. Physiol Rev.

[24] Yagishita S., Hayashi-Takagi A., Ellis-Davies G.C.R., Urakubo H., Ishii S., Kasai H. (2014). A critical time window for dopamine actions on the structural plasticity of dendritic spines. Science.

[25] Thum A.S., Gerber B. (2019). Connectomics and function of a memory network: the mushroom body of larval Drosophila. Curr Opin Neurobiol.

[26] Eichler K., Li F., Litwin-Kumar A., Park Y., Andrade I., Schneider-Mizell C.M., Saumweber T., Huser A., Eschbach C., Gerber B. (2017). The complete connectome of a learning and memory centre in an insect brain. Nature.

[27] Schroll C., Riemensperger T., Bucher D., Ehmer J., Völler T., Erbguth K., Gerber B., Hendel T., Nagel G., Buchner E., Fiala A. (2006). Light-induced activation of distinct modulatory neurons triggers appetitive or aversive learning in Drosophila larvae. Curr Biol.

[28•] Saumweber T., Rohwedder A., Schleyer M., Eichler K., Chen Y.-c., Aso Y., Cardona A., Eschbach C., Kobler O., Voigt A., Durairaja A., Mancini N., Zlatic M., Truman J.W., Thum A.S., Gerber B. (2018). Functional architecture of reward learning in mushroom body extrinsic neurons of larval Drosophila. Nat Commun.

[29] Aso Y., Hattori D., Yu Y., Johnston R.M., Iyer N.A., Ngo T.-T.B., Dionne H., Abbott L.F., Axel R., Tanimoto H., Rubin G.M. (2014). The neuronal architecture of the mushroom body provides a logic for associative learning. eLife.

[30] Fee M.S., Goldberg J.H. (2011). A hypothesis for basal ganglia-dependent reinforcement learning in the songbird. Neuroscience.

[31] Albus J.S. (1971). A theory of cerebellar function. Math Biosci.

[32] LeCun Y., Touresky D.S., Hinton G.E., Sejnowski T.J. (1988). A theoretical framework for back-propagation.. Proceedings of the 1988 Connectionist Models Summer School, vol 1.

[33] Bengio Y. (2014). How Auto-Encoders Could Provide Credit Assignment in Deep Networks Via Target Propagation. arxiv:/arXiv:1407.7906.

[34] Lillicrap T.P., Cownden D., Tweed D.B., Akerman C.J. (2016). Random synaptic feedback weights support error backpropagation for deep learning. Nat Commun.

[35] Lee D.-H., Zhang S., Fischer A., Bengio Y. (2015). Difference target propagation. Joint European Conference on Machine Learning and Knowledge Discovery in Databases.

[36] Liao Q., Leibo J.Z., Poggio T. (2016). How Important is Weight Symmetry in Backpropagation?. arxiv:/arXiv:1510.05067%20[cs].

[37] Whittington J.C.R., Bogacz R. (2017). An approximation of the error backpropagation algorithm in a predictive coding network with local Hebbian synaptic plasticity. Neural Comput.

[38] Meulemans A., Carzaniga F.S., Suykens J.A.K., Sacramento J., Grewe B.F. (2020). A Theoretical Framework for Target Propagation. arxiv:/arXiv:2006.14331%20[cs,%20stat].

[39] Nøkland A., Lee D., Sugiyama M., Luxburg U., Guyon I., Garnett R. (2016). Direct feedback alignment provides learning in deep neural networks. Advances in Neural Information Processing Systems, volume 29.

[40] Murray J.M. (2019). Local online learning in recurrent networks with random feedback. eLife.

[41] Bellec G., Scherr F., Subramoney A., Hajek E., Salaj D., Legenstein R., Maass W. (2020). A solution to the learning dilemma for recurrent networks of spiking neurons. Nat Commun.

[42] Payeur A., Guerguiev J., Zenke F., Richards B.A., Naud R. (2020). Burst-dependent synaptic plasticity can coordinate learning in hierarchical circuits. bioRxiv.

[43••] Bartunov S., Santoro A., Richards B.A., Marris L., Hinton G.E., Lillicrap T.P. (2018). Assessing the scalability of biologically-motivated deep learning algorithms and architectures. Advances in Neural Information Processing Systems 31.

[44] Kevin M.B., Boergens M., Helmstaedter M. (2015). SegEM: efficient image analysis for high-resolution connectomics. Neuron.

[45] Kornfeld J., Denk W. (2018). Progress and remaining challenges in high-throughput volume electron microscopy. Curr Opin Neurobiol.

[46] Motta A., Berning M., Boergens K.M., Staffler B., Beining M., Loomba S., Hennig P., Wissler H., Helmstaedter M. (2019). Dense connectomic reconstruction in layer 4 of the somatosensory cortex. Science.

[47] Schneider-Mizell C.M., Gerhard S., Longair M., Kazimiers T., Li F., Zwart M.F., Champion A., Midgley F.M., Fetter R.D., Saalfeld S. (2016). Quantitative neuroanatomy for connectomics in Drosophila. Elife.

[48] Zheng Z., Lauritzen J.S., Perlman E., Robinson C.G., Nichols M., Milkie D., Torrens O., Price J., Fisher C.B., Sharifi N. (2018). A complete electron microscopy volume of the brain of adult Drosophila melanogaster. Cell.

[49] Marr D. (1969). A theory of cerebellar cortex. J Physiol.

